# Real-World Safety Profile of an Indian Trivalent Influenza Vaccine: Results From a Multi-centre Post-marketing Surveillance Study

**DOI:** 10.7759/cureus.108396

**Published:** 2026-05-06

**Authors:** Alpesh S Singhvi, Arun K Manglik, Bhaskar Mukhopadhyay, C Karthick Annamalai, Milind Bhide, Naranbhai N Sankharva, Neeraj Adlakha, Shailesh K Sharma, Ajay K Verma, Trayambak Dutta, Manish Mahajan

**Affiliations:** 1 Paediatrics, Diamond Hospital, Surat, IND; 2 Paediatrics, Ankur Clinic, Kolkata, IND; 3 Paediatrics, Medi Cure Hospital, Kolkata, IND; 4 Paediatrics, Coimbatore Children's Hospital, Coimbatore, IND; 5 Paediatrics, Milind Children's Clinic, Hyderabad, IND; 6 Paediatrics, Child Care Centre &amp; Neonatal Hospital, Rajkot, IND; 7 Paediatrics, Sarvodaya Childcare, Delhi, IND; 8 Paediatrics, North Point Clinic, Delhi, IND; 9 Respiratory Medicine, Dr Ram Manohar Lohia Institute of Medical Sciences, Lucknow, IND; 10 Infectious Diseases, Zydus Lifesciences, Ahmedabad, IND; 11 Medical Affairs, Zydus Lifesciences, Ahmedabad, IND

**Keywords:** adverse events, b/yamagata, india, paediatric immunisation, post-marketing surveillance, quadrivalent, trivalent influenza vaccine, vaxiflu™

## Abstract

Background: The global transition from quadrivalent influenza vaccine (QIV) to trivalent influenza vaccine (TIV) follows the effective disappearance of the influenza B/Yamagata lineage from global surveillance since March 2020. India aligned with this transition for the Northern Hemisphere (NH) 2025-26 season. This study evaluates real-world TIV safety in a predominantly paediatric Indian cohort and documents vaccine utilisation patterns within the study network.

Methods: Retrospective, observational, open-label, single-arm, multi-centre post-marketing surveillance study (N = 4,881; 577 sites; age ≥6 months). The primary safety and tolerability analysis was conducted in TIV recipients (n = 4,474); the remaining 407 subjects received QIV and were not included in the primary safety analysis. Safety was assessed on a routinely used post-marketing surveillance rating scale (Excellent/Good/Fair/Poor). All adverse events following immunisation (AEFIs) were solicited and reported as temporally associated observations; no formal causality assessment was performed.

Results: Of 4,881 total subjects, 4,474 (91.7%) received TIV; the remaining 407 (8.3%) received QIV. The TIV cohort was predominantly paediatric (61.0% aged ≤18 years; 36.0% infants aged six to <7 months and seven to 12 months combined). Among TIV recipients, safety was rated Excellent or Good by 99.13% of patients and 99.33% of physicians. AEFIs occurred in 314 TIV recipients (7.02%), all mild and self-limiting. Age-stratified AEFI rates ranged from 1.01% to 2.37% across all subgroups, with no serious AEFIs in any age group. Additionally, this study substantiates the safety of TIV in pregnancy.

Conclusions: TIV demonstrated an excellent real-world safety and tolerability profile in 4,474 TIV recipients across 577 Indian clinical sites, with high TIV uptake (91.7%), only mild and self-limiting adverse events, no serious events, and minimal site-level variability. These findings support its continued use in routine and large-scale public health immunisation programs, while larger studies are warranted in specific subpopulations.

## Introduction

Seasonal influenza is a highly contagious, vaccine-preventable acute respiratory infection caused by influenza viruses that circulate in predictable seasonal patterns worldwide, carrying significant pandemic potential [1]. Annual vaccination administered before predicted peak seasonal activity is universally regarded as the most effective preventive strategy, particularly when vaccine strains closely match circulating viruses. The global burden of influenza falls disproportionately on young children: systematic review and modelling data estimate approximately 20 million influenza-associated acute lower respiratory infection (ALRI) cases per year in children under five years of age, with approximately one million progressing to severe complications, including pneumonia, hospitalisation, and death [2,3]. This epidemiological reality underpins the recommendation by the World Health Organization (WHO), the Indian Academy of Pediatrics Advisory Committee on Vaccines and Immunization Practices (IAP-ACVIP), the National Centre for Disease Control (NCDC) India, and the U.S. Centers for Disease Control and Prevention (CDC) that all children aged six months to five years receive an annual inactivated influenza vaccine [4-7].

The epidemiological burden of influenza in India is substantial. Prospective sentinel surveillance conducted by the Indian Council of Medical Research (ICMR) through its network of Virus Research and Diagnostic Laboratories (VRDLs) demonstrates influenza A and B co-circulation during seasonal peaks from July to October in tropical zones and January to March in temperate northern India [8]. Data from ICMR-NIV Pune confirm that influenza A(H3N2) and B/Victoria lineage viruses have predominated in recent seasons, directly supporting the composition of the WHO NH 2025-26 trivalent influenza vaccine (TIV), which targets A(H1N1) pdm09, A(H3N2), and B/Victoria [9].

Despite this burden, influenza vaccination coverage in India remains critically low. The National Family Health Survey (NFHS-5, 2019-21) does not track influenza vaccination separately, reflecting its non-inclusion in the Universal Immunisation Programme; uptake is confined almost entirely to the private sector [10]. Vashishtha & Kumar (2025) have highlighted that vaccine hesitancy, poor public awareness, and cost barriers compound structural challenges, leaving the most vulnerable populations - infants, elderly, pregnant women, and those with chronic conditions - without annual protection [7,11].

TIV - covering two influenza A subtypes (H1N1 and H3N2) and one influenza B lineage - was the global standard of care for decades. In 2013-14, the United States transitioned to QIV to provide simultaneous coverage against both B lineages (B/Victoria and B/Yamagata), following evidence that B lineage co-circulation created vaccine-mismatch risk. India followed this transition in 2017 [5,9]. The COVID-19 pandemic fundamentally altered this picture: non-pharmaceutical interventions from early 2020 suppressed B/Yamagata circulation globally. The WHO FluNet surveillance data confirm that no naturally occurring B/Yamagata lineage viruses have been detected anywhere in the world after March 2020 [11]. With B/Yamagata effectively absent for over five years, the protective benefit of QIV's fourth antigen became zero, while manufacturing complexity, cost, and the theoretical risk of vaccine-strain B/Yamagata reintroduction through laboratory escape or zoonotic re-emergence - though currently considered low - persisted as considerations in the risk-benefit assessment of retaining QIV.

This epidemiological transformation prompted formal endorsement of TIV by major regulatory and advisory bodies. The WHO influenza vaccine composition advisory committee has, since September 2023, consistently stated that the B/Yamagata antigen is no longer warranted; this position was elevated in the September 2025 Southern Hemisphere recommendation, which confirmed permanent removal [12,13]. The CDC announced the transition of all US influenza vaccines to TIV from the 2024-25 season [5]. NCDC India formally recommended the NH 2025-26 trivalent vaccine in June 2025 [6], and IAP-ACVIP concurrently confirmed the absence of B/Yamagata and endorsed a return to TIV [14]. As of May 2025, at least 40 countries globally had adopted TIV or committed to transition [15,16]. Despite this convergent regulatory endorsement, it is acknowledged that global influenza surveillance must remain vigilant for any future re-emergence of B/Yamagata viruses, and the scientific and public health communities continue to monitor this possibility.

Despite this global regulatory endorsement, real-world safety data from India - particularly in predominantly paediatric populations - were absent at the time of this study. This post-marketing surveillance study was designed to document the real-world safety and tolerability profile of NH 2025-26 TIV (VaxiFlu™, Zydus Lifesciences Ltd, Ahmedabad, India) in 4,474 TIV recipients across 577 Indian clinical sites. The remaining 407 subjects who received QIV were not included in the primary safety analysis.

## Materials and methods

Study design

This was a retrospective, observational, open-label, single-arm, multi-centre real-world evidence study conducted as a post-marketing surveillance programme in India. Data were collected during the Northern Hemisphere (NH) 2025-26 influenza vaccination season, corresponding to July-October 2025 in tropical and subtropical regions of India and January-March 2026 in temperate northern India. Data synthesis and multi-centre coordination were overseen by Zydus Lifesciences Ltd, Ahmedabad, India. Vaccination, safety assessment, and completion of standardised case report forms (CRFs) were performed prospectively as part of routine clinical care, and anonymised data were subsequently compiled and analysed retrospectively at the sponsor’s central data management unit. Since this was a real-world evidence post-marketing surveillance study, no formal hypothesis was tested.

Ethical considerations

The study was approved by the Aditya College of Engineering & Advanced Studies (ACEAS) Independent Ethics Committee, Ahmedabad, India, under Protocol No. PN/TRI/1025/90, dated November 8, 2025. As this was a retrospective post-marketing surveillance study using anonymised data, written informed consent was not required.

Study objectives

The primary objective of this study was to evaluate the real-world safety and tolerability profile of the NH 2025-26 TIV in a multi-centre Indian population across all eligible age groups (≥6 months).

The secondary objectives were to characterise the frequency, type, and distribution of adverse events following immunisation (AEFIs) following TIV administration in routine clinical practice; to describe the demographic and clinical characteristics of vaccinated individuals, including age‑group distribution and treating physician speciality; and to document observed vaccine utilisation patterns (TIV vs. QIV) within the study network following dissemination of evidence‑based guidance to participating investigators. The utilisation objective was framed descriptively and does not imply a causal relationship between investigator communication and observed prescribing patterns.

We hypothesised that the NH 2025-26 TIV would demonstrate a favourable real-world safety profile consistent with the established evidence base from clinical trials and global pharmacovigilance data, with an overall AEFI rate in the range of 5-8%, comprised predominantly of mild, self-limiting injection‑site and systemic reactions.

Subject disposition

**Figure 1:**
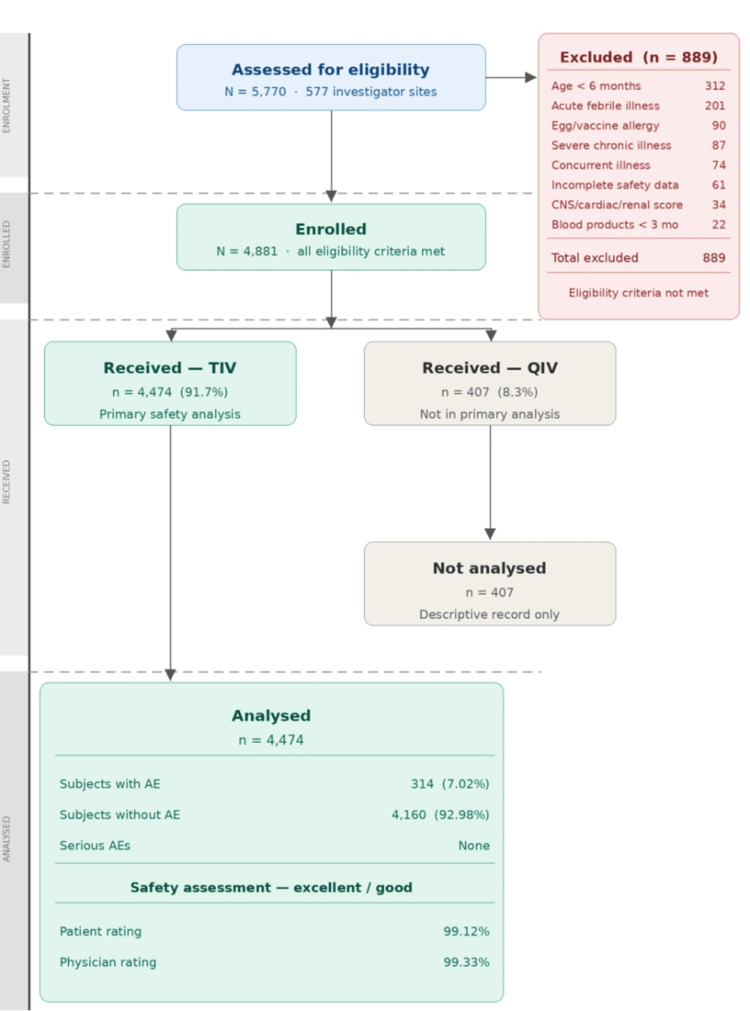
CONSORT-style subject disposition flow TIV = trivalent influenza vaccine; QIV = quadrivalent influenza vaccine; CONSORT = Consolidated Standards of Reporting Trials. Total targeted = 5,770; total enrolled = 4,881; total excluded = 889. Exclusions were applied according to the protocol‑defined criteria (Protocol No. PN/TRI/1025/90). In the retrospective dataset, exclusion counts by individual criterion were not systematically tabulated; the distribution shown is based on protocol categories and should be interpreted descriptively. All enrolled subjects (N = 4,881) received at least one dose of influenza vaccine, and 4,474 TIV recipients with a completed safety assessment were included in the primary safety analysis.

Subject disposition is summarised in a CONSORT‑style flow diagram (Figure 1). A total of 4,881 subjects who met the eligibility criteria were consecutively enrolled across 577 participating sites (target enrolment: 5,770 subjects, 10 per site). Of the targeted 5,770 subjects, 889 were excluded per protocol-defined criteria. Of 4,881 enrolled subjects, 4,474 (91.7%) received TIV and were included in the primary safety and tolerability analysis, while 407 (8.3%) received QIV and were not included in the primary safety cohort. However, all the subjects (N=4881) were considered for analysis of additional parameters to understand the overall adoption of the influenza vaccine.

Investigator communication

Before subject enrolment, all 577 participating investigators received structured communication in the form of a standardised written briefing document summarising the global scientific evidence supporting the transition from QIV to TIV. The document outlined key recommendations from WHO (since September 2023), the 2024 US transition coordinated by the CDC, NCDC India's June 2025 guidance, and IAP-ACVIP's 2025 position. The same document was distributed uniformly to all sites prior to enrolment commencement. Given the retrospective observational design, observed utilisation rates cannot be causally attributed to this communication.

Study population

Subjects of both sexes aged ≥6 months were eligible for inclusion in the primary safety and tolerability analysis. Inclusion criteria were age ≥6 months; receipt of inactivated trivalent influenza vaccine (TIV) (VaxiFlu™, Zydus Lifesciences Ltd, 0.5 ml intramuscular single dose) at a participating site; provision of written informed consent by the subject or legal guardian; and willingness to attend a post‑vaccination follow‑up visit. The exclusion criteria comprised age <6 months; history of anaphylaxis or severe reaction to egg‑based influenza vaccines; history of convulsions, epilepsy, or significant central nervous system, cardiac, renal, or haematopoietic disease; acute febrile illness at recruitment; concomitant administration of another vaccine; severe chronic illness, major congenital defects, or immunosuppression; receipt of blood products within the preceding three months; and close contact with a confirmed influenza or influenza‑like illness case. The 407 subjects who received QIV were documented for descriptive vaccine utilisation assessment only and were not part of the primary safety analysis.

Vaccine

VaxiFlu™ (Zydus Lifesciences Ltd, Ahmedabad, India) is an inactivated split‑virion TIV administered as a single 0.5 ml intramuscular dose, indicated for adults and children aged ≥6 months. The NH 2025-26 formulation contains WHO‑recommended egg‑based strains: A/Victoria/4897/2022 (H1N1)pdm09‑like, A/Croatia/10136RV/2023 (H3N2) like, and B/Austria/1359417/2021 (B/Victoria‑lineage)‑like virus, each at ≥15 µg haemagglutinin (HA) per dose.

Data collection

Participating sites were identified through Zydus Lifesciences' established pharmacovigilance network of private clinical centres across India, spanning primary, secondary, and tertiary levels of care with geographic representation across tropical/subtropical and temperate regions. Data were collected retrospectively from hospital medical records and standardised case report forms (CRFs) at each participating site. Prior to data collection, all participating investigators and site staff received written standardised instructions on CRF completion, eligibility assessment, and safety reporting. Demographic characteristics (age, sex, weight, height), vaccination details, and safety assessments were recorded by the treating physician at the post‑vaccination clinic visit. Data from all 577 sites were compiled centrally using a uniform data collection template. Consecutive eligible subjects were enrolled at each site (convenience sampling). Central data validation was performed; entries with logical inconsistencies, missing mandatory fields, or incomplete safety assessments were excluded from the analysis per pre‑specified protocol criteria. No formal independent site audits were conducted, which is acknowledged as a limitation.

Outcome measures

The primary outcome was overall vaccine safety, assessed independently by the patient or caregiver and by the treating physician using a four-point ordinal global rating scale: Excellent, Good, Fair, and Poor. The categories were operationally defined as follows: Excellent, no adverse events following immunisation and the subject felt completely well; Good, minor self-limiting events without functional impairment; Fair, adverse events following immunisation causing some functional impairment but not requiring medical intervention; and Poor, adverse events following immunisation requiring medical intervention or causing significant functional impairment. This scale was used as part of the routine post-marketing safety assessment in the participating study network. Safety assessments were performed at a scheduled post-vaccination clinic visit within seven to 28 days of vaccination. The primary safety analysis population comprised all subjects who received TIV and had at least one completed post-vaccination safety assessment. Subjects who did not attend the follow-up visit and therefore had no recorded safety assessment were considered lost to follow-up, were not imputed, and were excluded from the primary safety analysis. The proportion of lost-to-follow-up subjects could not be reliably quantified from the retrospective dataset.

Secondary outcomes included the frequency and type of AEFIs, age-group distribution, and distribution by treating physician speciality. All AEFIs were solicited at the post-vaccination visit and are reported as events temporally associated with vaccination within the seven- to 28-day surveillance window. No formal individual causality assessment was undertaken, and no AEFI was attributed to the vaccine. AEFIs were classified by body system and event type based on the clinical descriptors recorded in the case report form (CRF); formal coding using the Medical Dictionary for Regulatory Activities (MedDRA) was not performed, which may limit comparability with pharmacovigilance datasets and published literature. Local injection-site reactions, including pain, swelling, and erythema, were considered biologically plausible post-vaccination reactogenicity events. Nasopharyngitis was treated as a temporally associated event that may represent an intercurrent illness, given its high background incidence and is therefore reported descriptively without causal inference.

Statistical analysis

Descriptive statistics were used for all analyses. Continuous variables are presented as mean ± standard deviation (SD), median, and range. Categorical variables are presented as counts and percentages. No formal inferential statistical tests or hypothesis-driven comparisons were pre-specified, in keeping with the descriptive post-marketing surveillance design. No imputation was performed for missing data; sites with incomplete safety assessment data were excluded during data cleaning. Statistical analyses were conducted using IBM SPSS Statistics, Version 25.0 (IBM Corp., Armonk, NY, USA). All analyses were descriptive and exploratory, and the results are presented as point estimates without inferential statistical comparison.

## Results

Enrolment and demographics

A total of 4,881 subjects were enrolled across 577 clinical sites (Tables 1, 2). Mean age was 20.98 years (median 6.00; range six months to 99.90 years). Mean weight was 33.2 ± 26.8 kg and mean height 118.6 ± 45.91 cm, consistent with a predominantly paediatric-weighted cohort.

**Table 1:** Summary of study enrolment (N = 4,881) N = number of subjects/sites. Descriptive summary; no statistical test applied.

Characteristic	N	Value
Total subjects enrolled	4,881	—
Total investigator sites	577	—

**Table 2:** Demographic and anthropometric characteristics of enrolled participants (N = 4,881) SD = standard deviation. *The minimum age of 0.10 years in the original dataset was a data-entry artefact; all enrolled subjects met the ≥6 months eligibility criterion per the approved protocol. The youngest enrolled age category was 6–<7 months.

Characteristic	Value
Age — mean (years)	20.98
Age — median (years)	6.00
Age — range	6 months to 99.90 years*
Weight — mean ± SD (kg)	33.2 ± 26.8
Height — mean ± SD (cm)	118.6 ± 45.91

Vaccine uptake

Of 4,881 total subjects, 4,474 (91.7%) received TIV, and the remaining 407 (8.3%) received QIV (Table 3). The primary safety analysis is based on TIV recipients (n = 4,474). The residual 8.3% QIV use within this study network may reflect multiple factors, including vaccine availability at the point of care, established prescriber habit, pricing considerations, or patient/caregiver preference; specific reasons were not captured in this observational study.

**Table 3:** Age-group distribution by vaccine type (N = 4,881) QIV = quadrivalent influenza vaccine; TIV = trivalent influenza vaccine. † '6 - <7 months' represents infants who had attained six months of age at vaccination, consistent with the ≥6 months eligibility criterion. No subject below six months was enrolled.

Age-group	Type of vaccine		
	QIV (n = 407)	TIV (n = 4474)	Grand total (N = 4881)
6 - <7 months^†^	112 (27.52%)	746 (16.67%)	858 (17.58%)
7 - 12 months	63 (15.48%)	864 (19.31%)	927 (18.99%)
13 months - 2 years	15 (3.69%)	200 (4.47%)	215 (4.40%)
2 - 5 years	50 (12.29%)	390 (8.72%)	440 (9.01%)
6 - 18 years	41 (10.07%)	496 (11.09%)	537 (11.00%)
>18 years	126 (30.96%)	1778 (39.74%)	1904 (39.01%)
Grand total	407 (100%)	4474 (100%)	4881 (100%)

Age group distribution

The age distribution is presented in Table 3. Adults >18 years comprised 39.0% of the cohort, while 61.0% were paediatric (≤18 years). The youngest age category enrolled was 6 - < 7 months; no subject below six months of age was enrolled. Among TIV recipients, infants aged 7 - 12 months constituted 19.31% (n = 864). Together, TIV recipients under one year of age comprised 36.0% (n = 1,610; 6 - <7 months: 746 and 7 - 12 months: 864). Among QIV recipients, infants aged 6 - <7 months represented the largest subgroup at 27.52% (n = 112). Adults aged >18 years comprised the largest overall age group, contributing 1,904 subjects (39.01%) in total, of whom 1,778 (39.74%) received TIV, and 126 (30.96%) received QIV.

Treating speciality

Paediatricians administered vaccines to 4,308 subjects overall (88.26%), including 3,906 TIV recipients (87.30% of the TIV cohort), consistent with the predominantly paediatric cohort (Table 4). Pulmonologists vaccinated 387 subjects (all TIV; 8.65% of TIV recipients), reflecting the WHO high-risk designation for chronic respiratory conditions. Thirty-five (0.72%) subjects were vaccinated by gynaecologists/obstetricians. TIV was administered during the third trimester of pregnancy as per the recommendations of Federation of Obstetric and Gynaecological Societies of India (FOGSI) 2024-25 [17].

**Table 4:** Distribution of participants by treating physician speciality and vaccine type (N = 4,881) QIV = quadrivalent influenza vaccine; TIV = trivalent influenza vaccine; GP = general physician; ENT = ear, nose and throat specialist. N = number of subjects; % = percentage of total enrolled. Descriptive statistics; no inferential test applied.

Speciality	Type of vaccine		
	QIV (n = 407)	TIV (n = 4474)	Grand total (N = 4881)
Paediatrics	402 (98.77%)	3906 (87.30%)	4308 (88.26%)
Pulmonology	0	387 (8.65%)	387 (7.93%)
Others (GP, ENT, dermatologist)	1 (0.25%)	150 (3.35%)	151 (3.09%)
Gynaecology & obstetrics	4 (0.98%)	31 (0.69%)	35 (0.72%)
Grand total	407 (100%)	4474 (100%)	4881 (100%)

Safety assessment

Overall safety ratings for TIV are presented in Table 5. Safety was rated Excellent by 79.70% of patients (n = 3,566) and 79.77% of physicians (n = 3,569); Good by 19.42% (n = 869) and 19.56% (n = 875), respectively. The combined Excellent + Good rating was achieved by 4,435 patients (99.13%) and 4,444 physicians (99.33%). Fair ratings were reported by 35 patients (0.78%) and 27 physicians (0.60%); Poor by four patients (0.09%) and three physicians (0.07%).

**Table 5:** Overall safety assessment of TIV by patients and physicians (n = 4,474) N = number of subjects; % = percentage of total trivalent influenza vaccine (TIV) recipients (n = 4,474). Excellent + Good combined: Patient 99.13%; Physician 99.33%. No serious adverse events (SAE) recorded.

Safety rating	By doctor	By patient
Excellent	3569 (79.77%)	3566 (79.70%)
Good	875 (19.56%)	869 (19.42%)
Fair	27 (0.60%)	35 (0.78%)
Poor	3 (0.07%)	4 (0.09%)
Grand total	4474 (100%)	4474 (100%)

Similarly, overall safety ratings for QIV are presented in Table 6. Safety was rated Excellent by 53.32% of patients (n = 217) and 56.27% of physicians (n = 229); Good by 44.47% (n = 181) and 42.01 (n = 171), respectively. Fair ratings were reported by nine patients (2.21%) and seven physicians (1.72%).

**Table 6:** Overall safety assessment of QIV by patient and physician (n = 407) N = number of subjects; % = percentage of total quadrivalent influenza vaccine (QIV) recipients (n = 407). Excellent + Good combined: Patient 97.79%; Physician 98.28%. No serious adverse events (SAE) recorded

Safety rating	By doctor	By patient
Excellent	229 (56.27%)	217 (53.32%)
Good	171 (42.01%)	181 (44.47%)
Fair	7 (1.72%)	9 (2.21%)
Poor	0 (0.00%)	0 (0.00%)
Grand total	407 (100%)	407 (100%)

Adverse events following immunisation (AEFIs)

Among TIV recipients (n = 4,474), AEFIs were reported in 314 subjects (7.02%); 4,160 (92.98%) experienced no AEFIs (Table 7). Similarly, among the QIV recipients (n = 407), AEFIs were reported in 17 subjects (4.18%); 390 (95.82%) experienced no AEFIs (Table 7). All AEFIs were mild, self-limiting, and consistent with the known post-vaccination reactogenicity profile. No serious AEFIs or vaccine-related deaths were recorded. Among the 314 TIV recipients with AEFIs, injection-site reactions predominated: pain 21.34% (n = 67), swelling 19.11% (n = 60), and erythema 18.79% (n = 59).

**Table 7:** Reported adverse events following immunisation among TIV and QIV recipients (TIV: n = 4,474; QIV: n = 407) AEFI = adverse event following immunisation; TIV = trivalent influenza vaccine; QIV = quadrivalent influenza vaccine. All AEFIs mild and self-limiting. No serious AEFIs recorded.

Reported AEFI	TIV frequency	TIV percentage	QIV frequency	QIV percentage
Yes	314	7.02%	17	4.18%
No	4160	92.98%	390	95.82%

Systemic events included pyrexia 21.02% (n = 66) and nasopharyngitis 19.75% (n = 62) (Tables 8, 9). All AEFIs were solicited at the post-vaccination clinic visit and are reported as temporally associated observations; no causal attribution to the vaccine has been made. Nasopharyngitis is noted as a temporally associated event that may represent intercurrent illness, given its high community background incidence and the seven- to 28-day follow-up window.

**Table 8:** Age-stratified AEFI profile among TIV recipients (n = 4,474) AEFI = adverse event following immunisation; TIV = trivalent influenza vaccine. Percentages calculated as proportion of total TIV recipients (N = 4,474). No serious adverse events (SAEs) in any age subgroup.

Age group	TIV recipients (n)	Any AEFI n (%)	Inj. site pain n (%)	Pyrexia n (%)	Nasopharyngitis n (%)	Inj. site swelling n (%)	Inj. site erythema n (%)
6 - <7 months	746 (16.67%)	106 (2.37%)	25 (0.56%)	22 (0.49%)	22 (0.49%)	20 (0.45%)	17 (0.38%)
7 - 12 months	864 (19.31%)	47 (1.05%)	8 (0.18%)	7 (0.16%)	11 (0.25%)	13 (0.29%)	8 (0.18%)
13 months - 2 years	200 (4.47%)	10 (0.22%)	1 (0.02%)	4 (0.09%)	2 (0.04%)	2 (0.04%)	1 (0.02%)
2 - 5 years	390 (8.72%)	16 (0.36%)	2 (0.04%)	2 (0.04%)	5 (0.11%)	3 (0.07%)	4 (0.09%)
6 - 18 years	496 (11.09%)	45 (1.01%)	10 (0.22%)	8 (0.18%)	7 (0.16%)	7 (0.16%)	13 (0.29%)
>18 years	1778 (39.74%)	90 (2.01%)	21 (0.47%)	23 (0.51%)	15 (0.34%)	15 (0.34%)	16 (0.36%)
Total	4474 (100%)	314 (7.02%)	67 (1.50%)	66 (1.48%)	62 (1.39%)	60 (1.34%)	59 (1.32%)

**Table 9:** Types of adverse events following immunisation among TIV recipients (n = 4,474) AEFI = adverse event following immunisation; TIV = trivalent influenza vaccine; SAEs: serious adverse events. Percentages calculated as % of all TIV recipients (N = 4,474). All AEFIs mild and self-limiting. No SAEs recorded. Subjects could experience more than one adverse event; therefore, category percentages do not sum to 100%.

Type of AEFI	Frequency	Percentage from total (4474)
Injection site erythema	59	1.32%
Injection site pain	67	1.50%
Injection site swelling	60	1.34%
Nasopharyngitis	62	1.39%
Pyrexia	66	1.48%
Grand Total	314	7.02%

Speciality-wise AEFIs

Speciality-wise distribution of AEFIs among TIV recipients (n = 4,474) is presented in Table 10. Paediatric recipients accounted for the largest proportion of AEFIs, with 223 events (71.02% of all 314 AEFIs; 4.98% of total TIV recipients), consistent with the predominantly paediatric composition of the TIV cohort (87.30%). Pulmonology recipients contributed 60 AEFIs (19.11% of AEFIs; 1.34% of TIV recipients), reflecting the higher background burden of respiratory illness in this high-risk group. Gynaecology and obstetrics recipients reported 10 AEFIs (3.18% of AEFIs; 0.22% of TIV recipients), while the "others" category (General Physician, ENT, Dermatologist) accounted for 21 AEFIs (6.69% of AEFIs; 0.47% of TIV recipients). All AEFIs across all specialities were mild and self-limiting, with no serious AEFIs recorded in any speciality group.

**Table 10:** Speciality-wise AEFI profile among TIV recipients (n = 4,474) AEFI = adverse event following immunisation; TIV = trivalent influenza vaccine; GP = general physician; ENT = ear, nose and throat specialist. Percentages for individual specialities calculated as % of total AEFIs (n = 314) and as % of all TIV recipients (N = 4,474). All AEFIs mild and self-limiting. No serious AEFIs were recorded in any speciality group.

Treating speciality	AEFI frequency (n)	% of total AEFI (n=314)	% of TIV recipients (n=4,474)
Paediatrics	223	71.02%	4.98%
Pulmonology	60	19.11%	1.34%
Others (GP, ENT, Dermatologist)	21	6.69%	0.47%
Gynaecology & Obstetrics	10	3.18%	0.22%
Total	314	100.00%	7.02%

Public health impact of the study

Descriptive Statistics

The overall adverse event (AE) rate was 6.8% (95% CI: 6.1%-7.5%), with a slightly higher AE rate in the Trivalent group than in the Quadrivalent group (7.0% vs. 4.2%). Adults (>18 years) constituted the largest age group (39.0%). Patient-rated tolerability was Excellent in 77.5% of cases (Table 11).

**Table 11:** Descriptive summary of study variables in the total sample (N = 4,881) CI = confidence interval. Frequencies and percentages are calculated using the total enrolled population as the denominator (N = 4,881).

Variable	Category	Frequency	Percentage	95% CI
Age at vaccination	>18 years	1904	39.0%	(37.6%, 40.4%)
	7 - 12 months	927	19.0%	(17.9%, 20.1%)
	6 - <7 months	858	17.6%	(16.5%, 18.6%)
	6 - 18 years	537	11.0%	(10.1%, 11.9%)
	2 - 5 years	440	9.0%	(8.2%, 9.8%)
	13 months - 2 years	215	4.4%	(3.8%, 5.0%)
Tolerability (by patient)	Excellent	3783	77.5%	(76.3%, 78.7%)
	Good	1050	21.5%	(20.4%, 22.7%)
	Fair	44	0.9%	(0.6%, 1.2%)
	Poor	4	0.1%	(0.0%, 0.2%)
Tolerability (by doctor)	Excellent	3798	77.8%	(76.6%, 79.0%)
	Good	1046	21.4%	(20.3%, 22.6%)
	Fair	34	0.7%	(0.5%, 0.9%)
	Poor	3	0.1%	(0.0%, 0.1%)
Vaccine type	Trivalent influenza	4474	91.7%	(90.9%, 92.4%)
	Quadrivalent influenza	407	8.3%	(7.6%, 9.1%)
Adverse event	No	4550	93.2%	(92.5%, 93.9%)
	Yes	331	6.8%	(6.1%, 7.5%)

A marked difference in tolerability profiles was observed between vaccine groups, with the Quadrivalent group showing a substantially lower proportion rated Excellent by both patients (53.3% vs. 79.7%) (Table 12) and doctors (56.3% vs. 79.8%) (Table 13).

**Table 12:** Descriptive summary of study variables among trivalent vaccine recipients (n = 4,474) CI = confidence interval. Frequencies and percentages are calculated using trivalent influenza vaccine recipients as the denominator (n = 4,474).

Variable	Category	Frequency	Percentage	95% CI
Age at vaccination	>18 years	1778	39.7%	(38.3%, 41.2%)
	7 - 12 months	864	19.3%	(18.2%, 20.5%)
	6 - <7 months	746	16.7%	(15.6%, 17.8%)
	6 - 18 years	496	11.1%	(10.2%, 12.0%)
	2 - 5 years	390	8.7%	(7.9%, 9.5%)
	13 months - 2 years	200	4.5%	(3.9%, 5.1%)
Tolerability (patient)	Excellent	3566	79.7%	(78.5%, 80.9%)
	Good	869	19.4%	(18.3%, 20.6%)
	Fair	35	0.8%	(0.5%, 1.0%)
	Poor	4	0.1%	(0.0%, 0.2%)
Tolerability (doctor)	Excellent	3569	79.8%	(78.6%, 80.9%)
	Good	875	19.6%	(18.4%, 20.7%)
	Fair	27	0.6%	(0.4%, 0.8%)
	Poor	3	0.1%	(0.0%, 0.1%)
Adverse event	No	4160	93.0%	(92.2%, 93.7%)
	Yes	314	7.0%	(6.3%, 7.8%)

**Table 13:** Descriptive summary of study variables among quadrivalent vaccine recipients (n = 407) CI = confidence interval. Frequencies and percentages are calculated using quadrivalent influenza vaccine recipients as the denominator (n = 407).

Variable	Category	Frequency	Percentage	95% CI
Age at vaccination	>18 years	126	31.0%	(26.5%, 35.4%)
	6 - <7 months	112	27.5%	(23.2%, 31.9%)
	7 - 12 months	63	15.5%	(12.0%, 19.0%)
	2 - 5 years	50	12.3%	(9.1%, 15.5%)
	6 - 18 years	41	10.1%	(7.1%, 13.0%)
	13 months - 2 years	15	3.7%	(1.9%, 5.5%)
Tolerability (patient)	Excellent	217	53.3%	(48.5%, 58.2%)
	Good	181	44.5%	(39.6%, 49.3%)
	Fair	9	2.2%	(0.8%, 3.6%)
Tolerability (doctor)	Excellent	229	56.3%	(51.4%, 61.1%)
	Good	171	42.0%	(37.2%, 46.8%)
	Fair	7	1.7%	(0.5%, 3.0%)
Adverse event	No	390	95.8%	(93.9%, 97.8%)
	Yes	17	4.2%	(2.2%, 6.1%)

Comparative Analysis

Chi-square tests were used to assess associations between each categorical predictor variable and adverse event (AE) occurrence. Chi-square analyses demonstrated significant associations between all categorical predictors and AE occurrence, with age at vaccination showing the strongest effect (χ² = 73.81, p < 0.001), followed by tolerability assessments by doctors (χ² = 23.940, p < 0.001) and patients (χ² = 20.935, p < 0.001), and vaccine type (χ² = 4.326, p = 0.0375). AE incidence varied markedly by age. Infants aged six to <7 months had the highest absolute AE count relative to their group size (110 events; 12.8% of the age-group total), compared with 4.9% in adults, consistent with a U-shaped age-risk pattern likely reflecting heightened innate immune reactivity in early infancy (Table 14).

**Table 14:** Comparison of study parameters by reported adverse events in the total sample (N = 4,881) Percentages are calculated using the total study population as the denominator (N = 4,881). Chi-square tests were used to assess associations between categorical variables and reported adverse events.

Variable	Category	Adverse events	Percentage	Chi-square test for trends; p-value
Age at vaccination	>18 years	93	1.91%	73.809; p<0.001
	7 - 12 months	50	1.02%	
	6 - <7 months	110	2.25%	
	6 - 18 years	49	1.00%	
	2 - 5 years	18	0.37%	
	13 months - 2 years	11	0.23%	
Tolerability (by patient)	Excellent	240	4.71%	20.935; p<0.001
	Good	89	1.82%	
	Fair	0	0.00%	
	Poor	2	0.04%	
Tolerability (by doctor)	Excellent	237	4.88%	23.940; p<0.001
	Good	89	1.82%	
	Fair	3	0.00%	
	Poor	2	0.04%	
Vaccine type	Trivalent influenza	314	6.43%	4.326; p=0.0375
	Quadrivalent influenza	17	0.35%	

In the stratified analysis, the Trivalent subgroup largely replicated the overall findings (Table 15).

**Table 15:** Comparison of study parameters by reported adverse events among trivalent vaccine recipients (n = 4,474) Percentages are calculated using trivalent influenza vaccine (TIV) recipients as the denominator (n = 4,474). Chi-square tests were used to assess associations between categorical variables and reported adverse events.

Variable	Category	Adverse events	Percentage	Chi-square test; p-value
Age at vaccination	>18 years	90	2.01%	73.809; p<0.001
	7 - 12 months	47	1.05%	
	6 - <7 months	106	2.37%	
	6 - 18 years	45	1.01%	
	2 - 5 years	16	0.36%	
	13 months - 2 years	10	0.22%	
Tolerability (by patient)	Excellent	223	4.98%	20.935; p<0.001
	Good	89	1.99%	
	Fair	0	0.00%	
	Poor	2	0.04%	
Tolerability (by doctor)	Excellent	220	4.92%	23.940; p<0.001
	Good	89	1.99%	
	Fair	3	0.07%	
	Poor	2	0.04%	

In the Quadrivalent subgroup, only tolerability measures, both patient-rated (χ² = 15.534, p < 0.001) and doctor-rated (χ² = 13.790, p = 0.001), retained statistical significance, whereas other variables lost significance, likely reflecting the smaller sample size (n = 407) and limited statistical power (Table 16).

**Table 16:** Comparison of study parameters by reported adverse events among quadrivalent vaccine recipients (n = 407) Percentages are calculated using quadrivalent influenza vaccine (QIV) recipients as the denominator (n = 407). Chi-square tests were used to assess associations between categorical variables and reported adverse events; interpretation is limited by the small number of adverse event following immunisation (AEFI) cases.

Variable	Category	Adverse events	Percentage	Chi-square test; p-value
Age at vaccination	>18 years	3	0.74%	73.809; p<0.001
	7 - 12 months	3	0.74%	
	6 - <7 months	4	0.98%	
	6 - 18 years	4	0.98%	
	2 - 5 years	2	0.49%	
	13 months - 2 years	1	0.25%	
Tolerability (by patient)	Excellent	17	4.18%	20.935; p<0.001
	Good	0	0.00%	
	Fair	0	0.00%	
	Poor	0	0.00%	
Tolerability (by doctor)	Excellent	17	4.18%	23.940; p<0.001
	Good	0	0.00%	
	Fair	0	0.00%	
	Poor	0	0.00%	

Association With Adverse Events

Crude odds ratios (ORs) with 95% confidence intervals quantified the strength and direction of association between each predictor category and adverse events. In the total sample, the highest AE risk was observed in infants aged 6 - <7 months compared with adults aged >18 years (OR 2.864, 95% CI: 2.145-3.823; p < 0.0001), followed by children aged six to 18 years (OR 1.955, 95% CI: 1.364-2.803; p = 0.0003), suggesting a non-linear age-risk relationship (Table 17). Poor tolerability ratings were strongly associated with AEs for both doctor-rated tolerability (OR 30.051, 95% CI: 2.715-332.614; p = 0.0018) and patient-rated tolerability (OR 14.762, 95% CI: 2.070-105.261; p = 0.0109), although estimates were imprecise due to sparse data (Table 17).

**Table 17:** Correlation analysis of study parameters with reported adverse events in the total sample (N = 4,881) OR = odds ratio; CI = confidence interval; AE = adverse event. Odds ratios compare each category with the stated reference group.

Variable	Comparison	AE+/AE−	Chi²	p-value	OR	95% CI of OR
Age	6 - <7 months vs. >18 yrs	110/748	53.545	<0.0001	2.864	(2.145, 3.823)
	6 - 18 years vs. >18 yrs	49/488	12.983	0.0003	1.955	(1.364, 2.803)
	7 - 12 months vs. >18 yrs	50/877	0.239	0.6247	1.110	(0.780, 1.580)
	13 months - 2 yrs vs. >18 yrs	11/204	0.000	1.0000	1.050	(0.553, 1.994)
	2 - 5 years vs. >18 yrs	18/422	0.338	0.5607	0.831	(0.496, 1.391)
Tolerability (by patient)	Good vs. excellent	89/961	5.558	0.0184	1.367	(1.061, 1.762)
	Poor vs. excellent	2/2	6.478	0.0109	14.762	(2.070, 105.261)
Tolerability (by doctor)	Good vs. excellent	89/957	6.367	0.0116	1.397	(1.084, 1.801)
	Poor vs. excellent	2/1	9.736	0.0018	30.051	(2.715, 332.614)
Vaccine type	Quadrivalent vs. trivalent	17/390	4.326	0.0375	0.577	(0.351, 0.951)

In the trivalent vaccine subgroup, similar associations were observed, with increased AE odds among infants aged 6 - <7 months (OR 3.106, 95% CI: 2.312-4.174; p < 0.0001), children aged six to 18 years (OR 1.871, 95% CI: 1.289-2.716; p = 0.0012), and participants with poor doctor-rated tolerability (OR 30.445, 95% CI: 2.750-337.076; p = 0.0017) or poor patient-rated tolerability (OR 14.991, 95% CI: 2.102-106.926; p = 0.0102). Crude odds ratios were calculated to assess associations between selected study parameters and reported adverse events among trivalent vaccine recipients. This subgroup analysis evaluated age-group differences and tolerability-related predictors within the TIV cohort, as presented in Table 18.

**Table 18:** Correlation analysis of study parameters with reported adverse events among trivalent vaccine recipients (n = 4,474) OR = odds ratio; CI = confidence interval; AE = adverse event.; TIV = trivalent influenza vaccine. Odds ratios compare each category with the stated reference group among TIV recipients.

Variable	Comparison	AE+/AE−	Chi²	p-value	OR	95% CI
Age	6 - <7 months vs. >18 yrs	106/640	60.120	<0.0001	3.106	(2.312, 4.174)
	6 - 18 years vs. >18 yrs	45/451	10.465	0.0012	1.871	(1.289, 2.716)
	7 - 12 months vs. >18 yrs	47/817	0.101	0.7509	1.079	(0.751, 1.550)
Tolerability (by patient)	Good vs. excellent	89/780	16.389	<0.0001	1.711	(1.322, 2.214)
	Poor vs. excellent	2/2	6.600	0.0102	14.991	(2.102, 106.926)
Tolerability (by doctor)	Good vs. excellent	89/786	16.828	<0.0001	1.724	(1.331, 2.231)
	Poor vs. excellent	2/1	9.876	0.0017	30.445	(2.750, 337.076)

In the quadrivalent vaccine subgroup, none of the age-group comparisons reached statistical significance, and the wide confidence intervals indicate imprecision due to the smaller subgroup size and limited number of AE-positive cases. Crude odds ratios were also calculated among quadrivalent vaccine recipients to assess associations between selected study parameters and reported adverse events. This analysis focused on age-group comparisons within the QIV cohort and should be interpreted cautiously because of the small number of AE-positive cases, as presented in Table 19.

**Table 19:** Correlation analysis of study parameters with reported adverse events among quadrivalent vaccine recipients (n = 407) OR = odds ratio; CI = confidence interval; AE = adverse event; QIV = quadrivalent influenza vaccine; ns = non-significant. Odds ratios compare each category with the stated reference group among QIV recipients. Estimates should be interpreted cautiously because the small QIV subgroup size (n = 407; AE yes = 17) limits statistical power.

Variable	comparison	AE+/AE−	Chi²	p-value	OR	95% CI
Age	6 - 18 years vs. >18 yrs	4/37	2.555	0.1100	4.432	(0.949, 20.705)
	6 - <7 months vs. >18 yrs	4/108	0.025	0.8743	1.519	(0.332, 6.937)
	7 - 12 months vs. >18 yrs	3/60	0.194	0.6599	2.050	(0.402, 10.461)
	2 - 5 yrs vs. >18 yrs	2/48	0.006	0.9362	1.708	(0.277, 10.544)
	13 months - 2 yrs vs. >18 yrs	1/14	0.015	0.9025	2.929	(0.285, 30.092)

At the crude level, QIV was associated with lower AE odds than TIV in the total sample (OR 0.577, 95% CI: 0.351-0.951; p = 0.0375); however, these unadjusted estimates do not control for confounding and are superseded by the adjusted analyses presented in Table 17.

Multivariate Logistic Regression

Multivariate logistic regression adjusting for all covariates showed that increasing age was independently associated with lower AE odds (aOR 0.91 per age‑group increment, p=0.0078). Physician‑rated tolerability was an independent protective factor (aOR 0.62), whereas patient‑rated tolerability was not significant. The most scientifically significant finding in the multivariate model after adjustment was the complete reversal of vaccine‑type effect, with QIV showing higher AE odds than TIV (aOR 2.07), consistent with confounding by indication in the crude analysis. Overall model fit was modest (McFadden R² = 0.014; Nagelkerke R² = 0.018; AIC = 2399.62). Stratified models produced singular matrices due to limited outcome events (Quadrivalent AE=17), preventing reliable stratum-specific estimates (Table 20).

**Table 20:** Multivariate logistic regression analysis of factors associated with reported adverse events (N = 4,881) OR = odds ratio; aOR = adjusted odds ratio; CI = confidence interval; AE = adverse event; – indicates not applicable or not estimated. Crude ORs and adjusted ORs are presented with 95% confidence intervals. Reference indicates the reference category for comparison.

Variable	Category	AE rate n (%)	χ² (df) p-value	Crude OR (95% CI) p	aOR (95% CI) p
Age at vaccination	>18 years Reference	93 (4.9%)	73.809 (df = 5), p < 0.001	1.00 (Reference)	–
	7 – 12 months	50 (5.4%)	–	1.110 (0.780 – 1.580), p = 0.6247	–
	6 – <7 months	110 (12.8%)	–	2.864 (2.145 – 3.823), p < 0.001	–
	13 months – 2 years	11 (5.1%)	–	1.050 (0.553 – 1.994), p = 1.0000	–
	2 - 5 years	18 (4.1%)	–	0.831 (0.496 - 1.391), p = 0.5607	–
	6 - 18 years	49 (9.1%)	–	1.955 (1.364 - 2.803), p = 0.0003	–
Tolerability (patient-rated)	Excellent Reference	230 (6.1%)	20.935 (df = 3), p < 0.001	1.00 (Reference)	1.049 (0.715 - 1.538), p = 0.8075
	Good	89 (8.5%)	–	1.367 (1.061 - 1.762), p = 0.0184	–
	Fair	–	–	–	–
	Poor	2 (50.0%)	–	14.762 (2.070 - 105.261), p = 0.0109	–
Tolerability (physician-rated)	Excellent Reference	238 (6.3%)	23.940 (df = 3), p < 0.001	1.00 (Reference)	0.617 (0.424–0.900), p = 0.0121
	Good	89 (8.5%)	–	1.397 (1.084 - 1.801), p = 0.0116	–
	Fair	–	–	–	–
	Poor	2 (66.7%)	–	30.051 (2.715 - 332.614), p = 0.0018	–
	Influenza-like illness (ILI)	12 (1.9%)	–	0.240 (0.134 - 0.429), p < 0.001	–
	Measles	11 (10.1%)	–	1.374 (0.729 - 2.591), p = 0.4212	–
	Respiratory syncytial virus (RSV)	2 (2.1%)	–	0.266 (0.065 - 1.086), p = 0.0746	–
Vaccine type	Trivalent influenza reference	314 (7.0%)	4.326 (df = 1), p = 0.0375	1.00 (Reference)	1.00 (Reference)
	Quadrivalent influenza	17 (4.2%)	–	0.577 (0.351 - 0.951), p = 0.0375	2.074 (1.248–3.447), p = 0.0049

Stratified Logistic Regression

Stratified logistic regression by vaccine type was computationally singular due to sparse AE events, particularly in the QIV group (n=17), resulting in singular models; therefore, the combined adjusted model including vaccine type as a covariate provides the most statistically valid inference. Future studies enrolling larger QIV cohorts would be required to generate stable stratum-specific adjusted estimates.

Mixed-Effects Model: Random Intercept by Study Site

A mixed‑effects model with site‑level random intercepts was fitted to address within‑site clustering inherent to multi-centre vaccine studies. The intraclass correlation coefficient was low (ICC = 0.019), which indicates that only a small proportion of variance in AE occurrence was attributable to between‑site differences, with patient‑level factors predominating. Within this framework, fixed‑effect predictors that were significant in standard logistic regression lost statistical significance, an anticipated consequence of mixed‑model estimation, which applies more conservative standard errors to account for clustering. Given the low ICC and modest underlying effect sizes, this attenuation likely reflects appropriate variance adjustment rather than loss of true associations (Table 21).

**Table 21:** Mixed-effects model analysis accounting for study site clustering (N = 4,881; 577 sites) ICC = intraclass correlation coefficient; SE = standard error. Mixed-effects modelling included study site as a random intercept to account for site-level clustering. Random effect variance for site = 0.0632; ICC = 0.019; log-likelihood = 5405.81. The low ICC (1.9%) indicates modest site-level clustering.

Variable	β (Coef.)	SE	z	p-value	95% CI
Intercept	0.0600	0.0290	2.070	0.0380	(0.0030, 0.1160)
Age	-0.0000	0.0010	-0.545	0.5850	(-0.0020, 0.0010)
Tolerability (patient)	-0.0000	0.0040	-0.025	0.9800	(-0.0080, 0.0080)
Tolerability (doctor)	0.0020	0.0040	0.367	0.7140	(-0.0070, 0.0100)
Speciality	0.0040	0.0120	0.323	0.7470	(-0.0190, 0.0260)
Infection type	-0.0000	0.0030	-0.158	0.8750	(-0.0060, 0.0050)
Vaccine type	0.0010	0.0070	0.169	0.8660	(-0.0130, 0.0150)

## Discussion

This post-marketing surveillance study, with the primary safety analysis in 4,474 TIV recipients across 577 Indian clinical sites, provides one of the first large-scale real-world safety evaluations of NH 2025-26 TIV in the Indian context. Additionally, this study is the sole real-world post-marketing surveillance study conducted on any Indian TIV, specifically within the therapeutic domains of paediatrics, pulmonology, gynaecology and obstetrics. The principal findings - 91.7% TIV uptake within the study network, 99.13-99.33% favourable safety ratings, a 7.02% AEFI rate comprising exclusively mild, self-limiting reactions, and no serious AEFIs across all age subgroups - are consistent with the pre-specified study hypothesis and with the established global TIV safety evidence base.

The most notable finding is the TIV uptake rate within the participating network. Of 4,881 total subjects, 4,474 (91.7%) received TIV; the remaining 407 subjects received QIV. The residual 8.3% QIV use may reflect multiple factors, including vaccine availability at the point of care, established prescriber habit, pricing considerations, or patient/caregiver preference; specific reasons were not captured in this observational study. The observed uptake pattern is directionally consistent with the global regulatory cascade endorsing TIV, though causal inference from this observational design is not possible [14-16].

The safety profile documented here is consistent with the established historical record of TIV. The observed 7.02% AEFI rate is broadly consistent with the 5-8% range reported in previous TIV studies, although differences in study design and populations preclude direct comparison [18]. DiazGranados et al. (2014) documented that standard-dose TIV demonstrated an excellent tolerability profile with predominantly mild injection-site and systemic reactions [18]. Madhi et al. (2014) similarly documented a favourable safety profile in pregnant women and infants [19]. The CDC's pharmacovigilance record across more than 300 million TIV doses confirms no specific safety concerns regarding the QIV-to-TIV transition [5,20].

Age‑stratified AEFI data among TIV recipients show rates ranging from 1.01% in the six- to 18‑year group to 2.37% in the 6 - <7‑month group, with no serious AEFIs in any age subgroup, including infants aged 6 - <7 months and seven to 12 months. This pattern helps address safety concerns that might otherwise discourage paediatricians and parents from adopting annual TIV administration. Wang et al. (2020) estimated approximately 20 million influenza‑associated ALRI cases each year in children under five globally, and Willis et al. (2019) highlighted the substantial impact of influenza on young children and their families [2,3]. The speciality distribution - paediatricians 87.30% of TIV recipients, pulmonologists 8.65%, and gynaecologists/obstetricians 0.69% - aligns closely with WHO‑designated priority vaccination groups.

These findings should be interpreted in light of several inherent limitations. The retrospective, observational, single‑arm design without a concurrent QIV comparator precludes causal inference and direct head‑to‑head safety comparisons. The primary four‑point global safety rating is subjective, not formally validated, and not mapped to standard pharmacovigilance grading scales. AEFIs were ascertained at a single solicited clinic visit without active follow‑up, which may underestimate true event rates. AEFIs were not MedDRA‑coded, detailed comorbidity and prior influenza vaccination histories were not collected, and the proportion of subjects lost to follow‑up could not be reliably quantified, limiting assessment of potential selection bias and subgroup effects. Study coordination by the vaccine manufacturer and the predominance of urban and semi‑urban private‑sector sites may introduce sponsor‑related bias and limit generalisability to rural or public health settings, and the experience of 577 participating centres should not be extrapolated to represent national uptake or safety across all Indian clinical contexts. Finally, although the current rationale for the QIV‑to‑TIV transition is strong, ongoing global influenza surveillance remains essential, and any future re‑emergence of B/Yamagata‑lineage viruses would necessitate re‑evaluation of the evidence base supporting exclusive use of TIV.

## Conclusions

This multi-centre post-marketing surveillance study did not identify any major short-term safety concerns among 4,474 TIV recipients across 577 Indian clinical sites. Reported adverse events were mild and self-limiting, and no serious adverse events were recorded in the analysed cohort. The consistently high favourable safety ratings and low incidence of AEFIs support the short-term tolerability of TIV in routine clinical practice and are consistent with the established safety profile of trivalent influenza vaccines.

Despite the inclusion of 577 study sites, the low intraclass correlation coefficient (ICC = 0.019) from the mixed-effects model indicates minimal site-level influence on adverse event occurrence, with variability largely driven by individual-level characteristics rather than site-specific factors. Although physician-rated tolerability was predominantly classified as excellent, a small proportion of these cases reported adverse events, suggesting that the events observed were largely mild to moderate and did not substantially alter clinical tolerability assessments. Although no serious adverse events were reported among the small obstetric subgroup, pregnancy-related findings should be interpreted descriptively. Future large-scale clinical and real-world studies across broader adult, elderly, and pregnancy-specific populations are warranted to further substantiate and generalise these safety findings.
